# Sepsis prediction, early detection, and identification using clinical text for machine learning: a systematic review

**DOI:** 10.1093/jamia/ocab236

**Published:** 2021-12-13

**Authors:** Melissa Y Yan, Lise Tuset Gustad, Øystein Nytrø

**Affiliations:** Department of Computer Science, Faculty of Information Technology and Electrical Engineering, Norwegian University of Science and Technology, Trondheim, Norway; Department of Circulation and Medical Imaging, Faculty of Medicine and Health Sciences, Norwegian University of Science and Technology, Trondheim, Norway; Department of Medicine, Levanger Hospital, Clinic of Medicine and Rehabilitation, Nord-Trøndelag Hospital Trust, Levanger, Norway; Department of Computer Science, Faculty of Information Technology and Electrical Engineering, Norwegian University of Science and Technology, Trondheim, Norway

**Keywords:** sepsis, natural language processing, machine learning, electronic health records, systematic review

## Abstract

**Objective:**

To determine the effects of using unstructured clinical text in machine learning (ML) for prediction, early detection, and identification of sepsis.

**Materials and methods:**

PubMed, Scopus, ACM DL, dblp, and IEEE Xplore databases were searched. Articles utilizing clinical text for ML or natural language processing (NLP) to detect, identify, recognize, diagnose, or predict the onset, development, progress, or prognosis of systemic inflammatory response syndrome, sepsis, severe sepsis, or septic shock were included. Sepsis definition, dataset, types of data, ML models, NLP techniques, and evaluation metrics were extracted.

**Results:**

The clinical text used in models include narrative notes written by nurses, physicians, and specialists in varying situations. This is often combined with common structured data such as demographics, vital signs, laboratory data, and medications. Area under the receiver operating characteristic curve (AUC) comparison of ML methods showed that utilizing both text and structured data predicts sepsis earlier and more accurately than structured data alone. No meta-analysis was performed because of incomparable measurements among the 9 included studies.

**Discussion:**

Studies focused on sepsis identification or early detection before onset; no studies used patient histories beyond the current episode of care to predict sepsis. Sepsis definition affects reporting methods, outcomes, and results. Many methods rely on continuous vital sign measurements in intensive care, making them not easily transferable to general ward units.

**Conclusions:**

Approaches were heterogeneous, but studies showed that utilizing both unstructured text and structured data in ML can improve identification and early detection of sepsis.

## INTRODUCTION

Sepsis is a life-threatening illness caused by the body’s immune response to an infection that leads to multi-organ failure.^1^ Annually, there are 31.5 million sepsis cases, 19.4 million severe sepsis cases, and 5.3 million sepsis deaths estimated in high-income countries.^2^ Studies have shown that early identification of sepsis following rapid initiation of antibiotic treatment improves patient outcomes,^3^ and 6 h of treatment delay is shown to increase the mortality risk by 7.6%.^4^ Unfortunately, sepsis is commonly misdiagnosed and mistreated because deterioration with organ failure is also common in other diseases.^5–8^ The heterogeneity in infection source, immune responses, and pathophysiological changes make identification and therefore sepsis treatment difficult. Additionally, the diversity in age, gender, and comorbidities affect the symptoms and outcome of septic patients.^7^

Machine learning (ML) has been employed to improve sepsis outcomes through early detection. ML can utilize structured and unstructured data from electronic health records (EHRs).^9–14^ Structured clinical data come in a fixed format, such as age, vital signs, and laboratory data, which make data preprocessing easier. In contrast, clinical notes are in unstructured free-text form, such as progress notes, nursing notes, chief complaints, or discharge summaries. Clinical notes contain abbreviations, grammatical errors, and mis-spellings. Using clinical text is a complex, time-consuming process because it requires using natural language processing (NLP) to extract features that transform text into a machine-understandable representation.^15–22^ This usually requires assistance from clinical experts to convert text into machine-interpretable representations that capture clinical knowledge for specific clinical domains. The effort required to utilize unstructured clinical text can deter researchers; however, unstructured clinical text contains valuable information.^16^^,^^22–25^ Multiple studies and a review^25^ have shown that using unstructured clinical text has increased model performance to detect or predict colorectal surgical complications,^26^ postoperative acute respiratory failure,^27^ breast cancer,^28^ pancreatic cancer,^29^ fatty liver disease,^30^ pneumonia,^31^ inflammatory bowel disease,^32^^,^^33^ rheumatoid arthritis,^34–36^ multiple sclerosis,^37^ and acute respiratory infection.^38^^,^^39^

Prior reviews related to sepsis detection and prediction include: sepsis detection using Systemic Inflammatory Response Syndrome (SIRS) screening tools,^40^ sepsis detection using SIRS and organ dysfunction criteria with EHR vital signs and laboratory data,^41^ clinical perspectives on the use of ML for early detection of sepsis in daily practice,^14^ ML for diagnosis and early detection of sepsis patients,^9–13^ infectious disease clinical decision support,^42^ and healthcare-associated infections mentioning sepsis.^43–45^ However, to the best of our knowledge, no reviews focus on the effect of utilizing unstructured clinical text for sepsis prediction, early detection, or identification; this makes it challenging to assess and utilize text in future ML and NLP sepsis research.

## OBJECTIVE

The review aims to gain an overview of studies utilizing clinical text in ML for sepsis prediction, early detection, or identification.

## MATERIALS AND METHODS

This systematic review follows the Preferred Reporting Items for Systematic review and Meta-Analyses guidelines.^46^

### Search strategy

Relevant articles were identified from 2 clinical databases (PubMed and Scopus) and 3 computer science databases (ACM DL, dblp, and IEEE Xplore) using defined search terms. The 3 sets of search terms included: (1) “sepsis,” “septic shock,” or “systemic inflammatory response syndrome”; (2) “natural language processing,” “machine learning,” “artificial intelligence,” “unstructured data,” “unstructured text,” “clinical note,” “clinical notes,” “clinical text,” “free-text,” “free text,” “record text,” “narrative,” or “narratives”; and (3) detect, identify, recognize, diagnosis, predict, prognosis, progress, develop, or onset. Searches on clinical databases were performed using all 3 sets of search terms and excluded animal-related terms. Whereas searches on computer science databases only used the first set of search terms. No additional search restrictions, such as date, language, and publication status, were included. Additional articles were identified from relevant review articles or backward reference and forward citation searches of eligible articles. Complete search strategies are in Supplementary Table S1.

The search was initially conducted using only computer science databases on December 10, 2019 and was updated to include clinical databases on December 14, 2020. The first search found that 4 of 454 articles met inclusion criteria,^47–50^ and the second search uncovered 2 more articles that met inclusion criteria (6 of 1335 articles).^51^^,^^52^ Those 2 searches did not contain the search terms: “systemic inflammatory response syndrome,” “artificial intelligence,” identify, recognize, diagnosis, prognosis, progress, develop, and onset. Hence, a search on May 15, 2021, including those terms, found 2 additional articles.^53^^,^^54^ To ensure inclusion of other relevant articles, a broader search was conducted on September 3, 2021 to include the following terms: “unstructured data,” “unstructured text,” “clinical note,” “clinical notes,” “clinical text,” “free-text,” “free text,” “record text,” “narrative,” or “narratives.” This resulted in 1 additional article.^55^

### Study selection

Titles, abstracts, and keywords were screened using Zotero v5.0.96.3 (Corporation for Digital Scholarship, Vienna, VA) and Paperpile (Paperpile LLC, Cambridge, MA). Screening removed duplicates and articles that did not contain the following terms: (1) text, (2) notes, or (3) unstructured. Full-text articles were evaluated to determine if the study used unstructured clinical text for the identification, early detection, or prediction of sepsis onset in ML. Thus, selected articles had to rely on methods that automatically improve based on what they learn and not rely solely on human-curated rules. Additionally, articles solely focusing on predicting sepsis mortality were excluded as these articles are based on already established sepsis cases. Reviews, abstract-only articles, and presentations were removed. Additionally, a backward and forward search was performed on eligible full-text articles.

### Data extraction

One author independently extracted data, which a second author verified. Any discrepancies were resolved either through discussion with the third author by assessing and comparing data to evidence from the studies or by directly communicating with authors from included articles. The following information was extracted: (1) general study information including authors and publication year, (2) data source, (3) sample size, (4) clinical setting, (5) sepsis infection definition, (6) task and objective, (7) characteristics of structured and unstructured data, (8) underlying ML and NLP techniques, and (9) evaluation metrics.

## RESULTS

### Selection process

**Figure 1.**
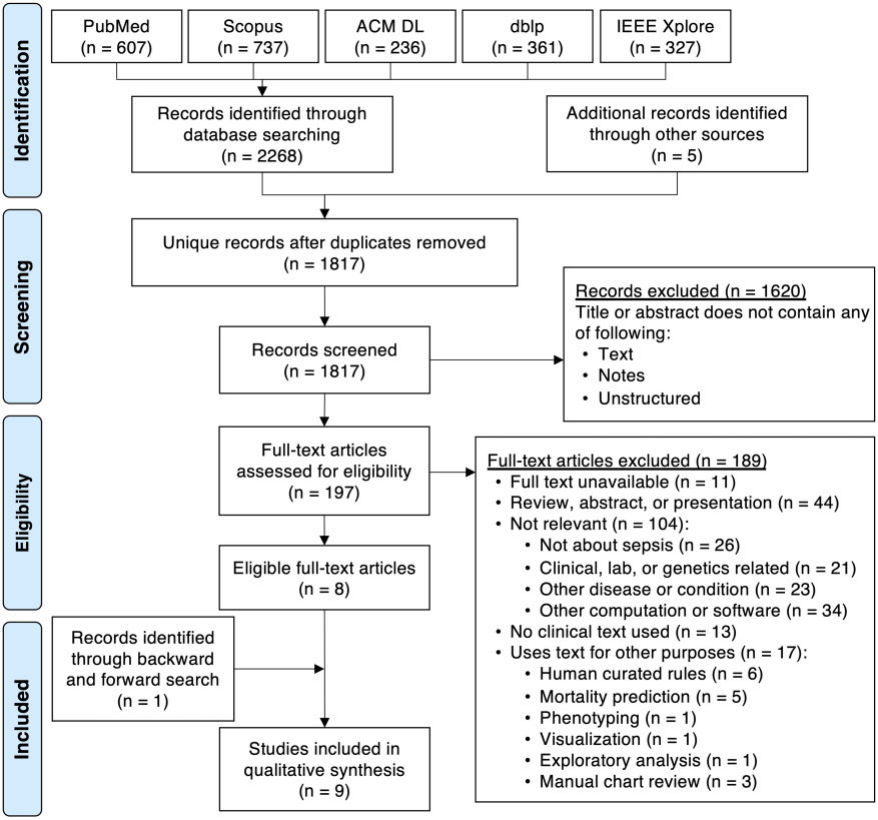
PRISMA (Preferred Reporting Items for Systemic reviews and Meta-Analyses) flowchart for study selection.

The initial search identified 2268 articles from 5 databases and 5 additional articles^56–60^ from 2 relevant review articles (Figure  1).^43^^,^^44^ From the 1817 unique articles, 1620 articles were excluded based on eligibility criteria described in the methods. After assessing the remaining 197 articles, most studies (189 of 197, ie, 96%) were excluded because they had not used or attempted to use unstructured clinical text in their ML models to identify, detect, or predict sepsis onset. For instance, there were sepsis-related studies that used text but for other purposes such as mortality prediction,^61–65^ phenotyping,^66^ visualization,^67^ exploratory data analysis,^68^ and manual chart review.^69–71^ Additionally, 6 articles about infection detection,^60^ central venous catheter adverse events,^58^ postoperative sepsis adverse events,^72–74^ and septic shock identification^75^ were excluded because they used manually human-curated rules instead of ML methods that automatically learn from data. The remaining 8 eligible articles were used to perform backward and forward searches,^47–50^^,^^52–55^ which led to the inclusion of 1 additional article.^51^ This resulted in 9 articles for synthesis.

### Study characteristics

Of the 9 identified articles, 2 studies aimed at identifying infection,^47^^,^^48^ 6 studies focused on early detection of sepsis,^51^^,^^53^^,^^55^ severe sepsis,^49^ or septic shock,^50^^,^^54^ and 1 study considered both identification and early detection for a combination of sepsis, severe sepsis, and septic shock.^52^ Most studies focused on intensive care unit (ICU)^48^^,^^50^^,^^52–55^ or emergency department (ED)^47^^,^^51^ data; only 1 used inpatient care data.^49^ Four studies utilized data from hospitals,^47^^,^^49^^,^^51^^,^^52^ 1 utilized MIMIC-II^54^ and 4 utilized MIMIC-III.^48^^,^^50^^,^^53^^,^^55^ MIMIC-II and MIMIC-III are publicly available ICU datasets created from Boston’s Beth Israel Deaconess Medical Center; MIMIC-II contains data from 2001–2007^76^ and MIMIC-III contains data from 2001–2012.^77^ Eight studies used data from the United States^47–51^^,^^53–55^ and 1 study used data from Singapore.^52^ Sample sizes varied greatly in terms of the number of patients or notes used. To select patient cohorts or notes associated with sepsis, 3 studies used International Statistical Classification of Diseases and Related Health Problems (ICD) codes,^47^^,^^49^^,^^52^ 5 applied sepsis definition criteria,^49–51^^,^^53^^,^^55^ 1 utilized descriptions of antibiotics usage,^48^ and another^54^ applied criteria from Henry et al^78^ that include ICD codes, sepsis criteria, and notes mentioning sepsis or septic shock. Table  1 summarizes the study characteristics and additional details are in Supplementary Table S2 (for Culliton et al,^49^ the 8 structured variables for the Modified Baystate clinical definition of severe sepsis and 29 structured variables used in models were provided through personal communications with the corresponding author of Culliton et al,^49^ Steve Gallant, on June 4, 2021).

**Table 1.** Study characteristics

Study (year)	Clinical setting and data source	Sample size^a^	Cohort criteria infection definition	Task and objective
Horng et al.^47^ (2017)	ED Beth Israel Deaconess (Boston, MA, United States) Dec 17, 2008—Feb 17, 2013	230 936 patient visits Infection: 32 103 P; 14% No infection: 198 833 P; 86% Train : 147 799 P; 64% Validation: 46 187 P; 20% Test: 36 950 P; 16%	Angus Sepsis ICD-9-CM abstraction criteria^79^	Identify patients with suspected infection to demonstrate benefits of using clinical text with structured data for detecting ED patients with suspected infection.
Apostolova and Velez^48^ (2017)	ICU MIMIC-III 2001–2012	634 369 nursing notes Infection presence: 186 158 N; 29% Possible infection: 3262 N; 1% No infection: 448 211 N; 70% Train: 70% Test: 30%	Notes describing patient taking or being prescribed antibiotics for treating infection	Identify notes with suspected or presence of infection to develop a system for detecting infection signs and symptoms in free-text nursing notes.
Culliton et al.^49^ (2017)	Inpatient care Baystate hospitals (Springfield, MA, United States) 2012–2016	203 000 adult inpatient admission encounters Used 68 482 E Severe sepsis: 1427 E; 2.1% 3-fold cross validation: only text data Model construction: 2012–2015 data Test set: 2016 data: Used 13 603 E Severe sepsis: 425 P; 3.1%	Modified Baystate clinical definition of severe sepsis (8 structured variables) and severe sepsis ICD codes	Predict severe sepsis 4, 8, and 24 h before the earliest time structured variables meet the severe sepsis definition to compare accuracy of predicting patients that will meet the clinical definition of sepsis when using unstructured data only, structured data only, or both types.
Delahanty et al.^51^ (2019)	ED Tenet Healthcare Hospitals (Nashville, TN, United States) January 1, 2016—October 31, 2017	2 759 529 patient encounters Sepsis: 54 661 E; 2% No Sepsis: 2 704 868 E; 98% Train: 1 839 503 E; 66.7% Sepsis: 36 458 E; 2% No sepsis: 1 803 045 E; 98% Test: 920 026 E; 33.3% Sepsis: 18 203 E; 2% No sepsis: 901 823 E; 98%	Rhee’s modified Sepsis-3 definition^80^	Predict sepsis risk in patients 1, 3, 6, 12, and 24 h after the first vital sign or laboratory result is recorded in the EHR to develop a new sepsis screening tool comparable to benchmark screening tools.
Liu et al.^50^ (2019)	ICU MIMIC-III 2001–2012	38 645 adult patients Train: 70% P Test: 30% P Applied model to: 15 930 P with suspected infection and at least 1 physiological EHR data	Sepsis-3 definition^1^	Predict septic shock in sepsis patients before the earliest time septic shock criteria are met to demonstrate an approach using NLP features for septic shock prediction.
Amrollahi et al.^53^ (2020)	ICU MIMIC-III 2001–2012	40 175 adult patients Sepsis: 2805 P; ∼7% Train 80% P Test 20% P	Sepsis-3 definition^1^	Predict sepsis onset hours in advance using a deep learning approach to show a pre-trained neural language representation model can improve early sepsis detection.
Hammoud et al.^54^ (2020)	ICU MIMIC-II 2001–2007	17 763 patients Sepsis: 6097 P Severe sepsis: 3962 P Septic shock : 1469 P 5-fold cross validation	Sepsis definition based on what Henry et al^78^ used	Predict early septic shock in ICU patients using a model that can be optimized based on user preference or performance metrics.
Goh et al.^52^ (2021)	ICU Singapore government-based hospital (Singapore, Singapore) Apr 2, 2015—Dec 31, 2017	5317 patients (114 602 notes) Train and validation: 3722 P (80 162 N) Sepsis: 6.45% No sepsis: 93.55% Test: 1595 P (34 440 N) Sepsis: 5.45% No sepsis: 94.55%	ICU admission with an ICD-10 code for sepsis, severe sepsis, or sepsis shock	Identify if a patient has sepsis at consultation time or predict sepsis 4, 6, 12, 24, and 48 h after consultation to develop an algorithm that uses structured and unstructured data to diagnose and predict sepsis.
Qin et al.^55^ (2021)	ICU MIMIC-III 2001–2012	49 168 patients Train: 33 434 P Sepsis: 1353 P No Sepsis: 32 081 P Validation: 8358 P Sepsis: 338 P No Sepsis: 8020 P Test: 7376 P Sepsis: 229 P No Sepsis: 7077 P	PhysioNet Challenge restrictive Sepsis-3 definition^81^	Predict if a patient will develop sepsis to explore how numerical and textual features can be used to build a predictive model for early sepsis prediction.

ED: emergency department; ICU: intensive care unit; ICD: International Classification of Diseases; ICD-9 CM: ICD Clinical Modification, 9th revision; ICD-10: ICD 10th revision; MIMIC-II: Multiparameter Intelligent Monitoring in Intensive Care II database; MIMIC-III: Medical Information Mart for Intensive Care dataset.

a Sample size unit abbreviations: P: patients; N: notes; E: encounters.

### Clinical text used in models

The 9 studies utilized narrative notes written by nurses,^47–50^^,^^53–55^ physicians,^49–53^^,^^55^ or specialists^49–51^^,^^54^^,^^55^ to document symptoms, signs, diagnoses, treatment plans, care provided, laboratory test results, or reports. EHRs contain various types of clinical notes. A note covers an implicit time period or activity and describes events, hypotheses, interventions, and observations within the health care provider’s responsibilities. The note’s form depends on its function: an order, a plan, a prescription, an investigation or analysis report, a narrative or log of events, information for the next shifts, or a requirement for legal, medical, or administrative purposes. An episode of care begins when a patient is admitted to the hospital and ends when the patient is discharged. Throughout a patient’s hospital stay, documentation can include chief complaints, history-and-physical notes, progress notes, reports, descriptions of various laboratory tests, procedures, or treatments, and a discharge summary. Chief complaints are the symptoms or complaints provided by a patient for why they are seeking care.^82^ History-and-physical notes can include history about the current illness, medical history, social history, family history, a physical examination, a chief complaint, probable diagnosis, and a treatment plan.^83^ Progress notes document care provided and a description of the patient’s condition to convey events to other clinicians.^84^ Free-text reports can include interpretations of echocardiograms, electrocardiograms (ECGs), or imaging results such as X-rays, computerized tomography scans, magnetic resonance imaging scans, and ultrasounds. At discharge, the health care personnel write a discharge summary note comprised of patient details, hospital admittance reason, diagnosis, conditions, history, progress, interventions, prescribed medications, and follow-up plans.^85–87^ The discharge summary letter is a formal document used to transfer patient care to another provider for further treatment and follow-up care.^88–90^

Studies have shown that nursing documentation differs from physician documentation.^91^^,^^92^ Nurses document more about a patient’s functional abilities than physicians,^91^ and the information from notes used and the frequency of viewing and documenting differs between health care personnel.^92^ Additionally, documentation varies between hospitals,^93^^,^^94^ hospitals have different resources and practices,^95–97^ and communicative behavior differs among professions in different wards.^98^ Hence, the type of notes used, who wrote the notes, and purpose of the note will play a role in how the documentation is interpreted.^99^

**Figure 2.**
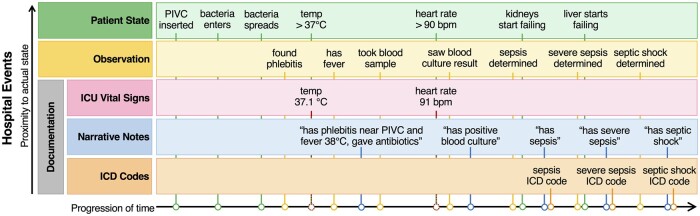
Overview of data from a patient timeline used to create models. The proximity of events toward a patient’s actual state and the actual documentation recorded in the electronic health records typically has delays. Green represents patient states as sepsis develops in a patient. Yellow are observations made by clinicians. Documentation includes ICU vital signs^a^ in pink, narrative notes in blue, and ICD codes in orange. ICU vital sign^a^ documentation can be instantaneous, narrative notes can be written after observations are made, and ICD codes are typically registered after a patient is discharged. PIVC: peripheral intravenous catheter. ^a^Vital signs include temperature, pulse, blood pressure, respiratory rate, oxygen saturation, and level of consciousness and awareness.

Table  2 provides information regarding documentation types, author of the note, time content of the data, time latency between documentation and availability in records, and the documentation frequency. In Figure  2, the relationship between hospital events and longitudinal data used to train models is shown. As sepsis develops in a patient over time, it shows there are typically delays between a patient’s actual state, clinical observations, and recorded documentation, such as ICU vital signs, narrative notes, and ICD codes.

**Table 2.** Clinical documentation from electronic health records

Documentation types	Author	Description	Temporal perspective	Record latency^a^	Frequency
Chief complaints	Physician Nurse Specialist	Symptoms or complaints provided by a patient at start of care for why they are seeking care.	Current	Seconds to days	One per episode
History-and-physical notes	Physician Nurse	Past medical history, family history, developmental history of present illness, problems about present illness, past medications or immunizations, allergies, or habits.	Retrospective	Immediately	One per episode
Progress notes	Physician Nurse Specialist (eg, respiratory therapist)	Observations of patient status and care provided to document progress and response to treatment plans. For physician, it includes determining diagnosis, prescriptions, and laboratory orders.	Retrospective Prospective	4–8 h	One per shift
Reports	Specialist	Radiologist results and cardiology results.	Retrospective	Days	One to many per episode
Discharge summary notes	Health care personnel	Episode of care summary and follow-up plans.	Retrospective Prospective	At discharge or days after	One per episode
Discharge summary letter	Physician	Formal required letter containing follow-up treatment plans.	Retrospective Prospective	Days to months after episode	One per episode
Laboratory results	Laboratory technician	Laboratory test analysis results from provided samples (eg, blood, urine, skin, and device) based on the physician’s order.	Retrospective	Days	One to many per episode
ICD codes	Physician Professional ICD coder ICD data aggregator organization	Diagnosis classification for billing.	Retrospective	Days to months	One per episode
Administrative	Administration	Patient information such as name, age, gender, address, contact information, and occupation.	Retrospective Current	Immediately	One per episode

a Record latency is defined as time between measurement/observation and the availability of the results in electronic health records.

The included studies utilized the following types of notes: 6 studies used unstructured nursing-related documentation,^47^^,^^48^^,^^50^^,^^53–55^ 4 used physician notes,^50^^,^^52^^,^^53^^,^^55^ 3 used radiology reports,^50^^,^^54^^,^^55^ 3 used respiratory therapist progress notes,^50^^,^^54^^,^^55^ 2 used ED chief complaints,^47^^,^^51^ 2 used ECG interpretations,^50^^,^^54^ 2 used pharmacy reports,^50^^,^^54^ 2 used consultation notes,^50^^,^^52^ 1 used discharge summaries,^50^ 1 included mostly progress notes and history-and-physical notes,^49^ and 3 used additional unspecified notes.^49^^,^^50^^,^^54^ Not all notes used are listed. Liu et al^50^ used all MIMIC-III notes to build a vocabulary of unique words, and discharge summaries were likely not used in predictions because they are unlikely to occur before observations. Additionally, Hammoud et al^54^ used all MIMIC-II notes except discharge summaries.

**Figure 3.**
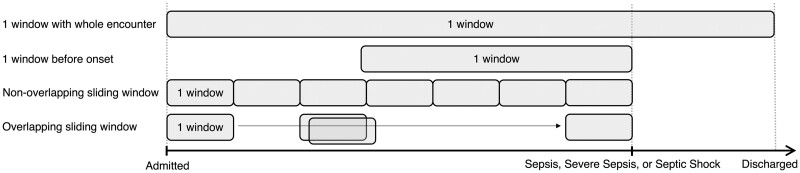
Different types of windows were used to obtain longitudinal data. Each gray box represents a single window, which can vary in duration (length of time) depending on the study. One window with the whole encounter means the study used a single window containing data with a duration of the whole encounter from admittance until discharge. One window before onset signifies data from a window with a duration of time before sepsis, severe sepsis, or septic shock onset. Sliding windows are consecutive windows until before sepsis, severe sepsis, or septic shock onset; this includes non-overlapping and overlapping sliding windows. Non-overlapping sliding windows indicate that data within one window of a fixed duration does not contain data in the next window. In contrast, overlapping sliding windows indicate windows of a fixed duration overlap, and data within one window will be partially in the next window.

These 9 studies utilized clinical notes differently. For the unit of analysis, 6 studies used a single note,^47^^,^^48^^,^^50^^,^^52–54^ 1 used a set of many notes from a patient encounter,^49^ 1 used a set of many notes within a specific hour of consideration,^55^ and 1 used keywords from notes.^51^ To identify infection signs, Horng et al^47^ and Apostolova and Velez^48^ processed individual notes. While Goh et al^52^ used notes at each patient consultation instance to identify sepsis patients. For early detection, 5 studies defined onset time as the earliest time when definition criteria are met^49^^,^^50^^,^^53–55^ and 1 defined sepsis onset time as ICU ward admission time.^52^ Studies for early detection used varying windows with different durations. A window decides how and where to obtain longitudinal data, and duration is the length of time. As shown in Figure  3, studies can use windows differently, such as a window with the duration of the whole encounter, a window with a duration of hours before onset, non-overlapping sliding windows with a fixed duration until onset, or overlapping sliding windows with a fixed duration until onset. Culliton et al^49^ used a 4-, 8-, or 24-h duration window before severe sepsis, and concatenated all text within a window. Goh et al^52^ used a 4-, 6-, 12-, 24-, or 48-h duration window of before sepsis, severe sepsis, or septic shock onset. Liu et al^50^ used 10 data points within a 1-h duration window spanning 2 h before septic shock, and used the most recently entered note for a data point to predict septic shock. Hammoud et al^54^ binned data in 15-minute duration non-overlapping sliding windows to update septic shock predictions every 15 minutes, and used the last note within the window. Amrollahi et al^53^ binned data into 1-h duration non-overlapping sliding windows to provide hourly sepsis predictions, and used sentences within a note to capture the semantic meanings. Qin et al^55^ used 6-h duration overlapping sliding windows with 6 data points to predict sepsis; a data point was generated from each hour within the window and all clinical notes within the hour were concatenated in random-order. Delahanty et al^51^ used a 1-, 3-, 6-, 12-, or 24-h duration window after the first vial sign or laboratory result was documented in the EHR to identify patients at risk for sepsis, and utilized keywords.

**Figure 4.**
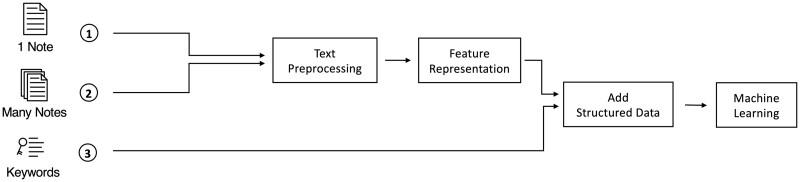
The unit of analysis used to train machine learning models for the included studies was either (1) a single note, (2) a set of many notes, or (3) keywords. In general, text was preprocessed and represented as features interpretable by a computer, then structured data were added, and the data were used to fit machine learning models.

First 2 columns in Table  3 show the type of text and unit of analysis used. Additional details about variables and specific notes used are listed in Supplementary Table S3 (the types of notes and usage for Liu et al^50^ was confirmed through personal communications with Ran Liu on June 2, 2021, for Hammoud et al^54^ by Ibrahim Hammoud on May 29, 2021, and for Qin et al^55^ by Fred Qin on September 9, 2021. Additionally, the structured variables used in models for Culliton et al^49^ were provided through personal communications with Steve Gallant on June 4, 2021). In Figure  4, single notes or a set of many notes are preprocessed and represented to extract features, whereas keywords are used as is. Then structured data can be added, and the data are used to train ML models.

**Table 3.** Text used in studies

Study (year)	Free-text document type	Unit of analysis	Text processing
Horng et al.^47^ (2017)	ED chief complaints Nursing triage assessments	One note	Representation: Bi-gram BoW (15 240-word vocabulary) LDA topic modeling (500 topics) Techniques: Convert to lowercase Remove rare tokens and punctuation Negation
Apostolova and Velez^48^ (2017)	Nursing notes	One note	Representation: BoW CBOW (200 vector size with window size of 7 = 441-term vocabulary of antibiotics usage and rules for negation and speculations) tf-idf PV (600 vector size for document-level representation) Techniques: Convert to lowercase Remove frequent tokens and non-alphanumeric characters Negation
Culliton et al.^49^ (2017)	Clinical notes (mostly progress notes and history-and-physical notes)	One patient encounter = many notes	Representation: GloVe (300-dimensional vector) + summing word vectors Techniques: Concatenated all notes for an encounter into a single text block
Delahanty et al.^51^ (2019)	ED chief complaints	Keywords	Other: Keywords extracted by experts
Liu et al.^50^ (2019)	All MIMIC-III clinical notes, such as but not limited to: Nursing notes Physician notes	One note	Representation: BoW (8907 unique term vocabulary and 832 predictive terms) GloVe (300-dimensional vector for each unique term) Techniques: Convert to lowercase Remove rare tokens, frequent tokens, and non-alphanumeric characters
Amrollahi et al.^53^ (2020)	Nursing notes Physician notes	One note	Representation: tf-idf (2227 vector size features = 2187 text features + 40 structured features) ClinicalBERT (808 vector size features = 768 text features + 40 structured features) Techniques: Remove rare tokens, frequent tokens, stop words, dates, and special characters
Hammoud et al.^54^ (2020)	All MIMIC-II notes except discharge summaries, such as but not limited to: Nursing progress notes Respiratory therapist progress notes	One note	Representation: BoW tf-idf Techniques: Remove rare and frequent tokens
Goh et al.^52^ (2021)	Physician notes: Admission notes Progress notes ICU consultations Pharmacy notes Allied health notes	One note	Representation: tf-idf LDA topic modeling (100 topics) Techniques: Remove rare tokens, punctuation, and stop words Lemmatization POS tagging Manual classification of topics into categories
Qin et al.^55^(2021)	Nursing notes Physician notes Radiology notes Respiratory notes	Many notes	Representation: tf-idf (1000 vector size = 1000 most common term vocabulary) ClinicalBERT (768 vector size features^a^ = either by concatenating all text first as input or using individual notes as input and concatenating output of individual notes) Techniques: Random-order concatenation of all clinical notes within the hour of consideration.^a^ Named entity recognition

BoW: Bag-of-words; CBOW: Continuous bag-of-words; ClinicalBERT: Clinical Bidirectional Encoder Representations from Transformers; ED: emergency department; GloVe: Global Vectors for Word Representation; ICU: intensive care unit; LDA: Latent Dirichlet Allocation; POS tagging: Part-of-speech tagging; PV: paragraph vectors; tf-idf: term frequency-inverse document frequency.

a Representation and technique details for Qin et al^55^ were provided through personal communications (with Fred Qin on September 7, 2021).

As shown in Figures  3 and 4 and listed in Tables  1 and 3 and Supplementary Tables S2 and S3, although all studies are related to sepsis, there are varying sample sizes, data types, inclusion criteria, and objectives. This heterogeneity makes it challenging to compare results for a meta-analysis.

### Natural language processing and machine learning study outcomes

To utilize text in ML, it must be transformed into a representation understandable by computers. In order to do that, Bag-of-words (BoW),^100^ n-gram, term frequency-inverse document frequency (tf-idf), and paragraph vectors (PV)^101^ representations can be used. These representations can be improved using additional NLP techniques, such as stop word removal, lemmatization, and stemming. In addition, other useful features can be extracted from text using part-of-speech (POS) tagging, named entity recognition, or Latent Dirichlet Allocation (LDA) topic modeling.^102^ In recent years, neural networks (NNs) have shown high predictive performance. As a result, many state-of-the-art results have been achieved using NNs to learn a suitable representation of texts, often known as embeddings.^103^ Embedding techniques include Global Vectors for Word Representation (GloVe),^104^ Word2Vec as a continuous bag-of-words (CBOW) model or skip-gram model,^105^ Bidirectional Encoder Representations from Transformers (BERT),^106^ and ClinicalBERT.^107^ The advantage of using embeddings is that it retains the sequential information lost in a BoW representation and does feature extraction automatically.^103^

Utilized text processing operations are in Table  3. One study used keyword extraction instead of text processing operations.^51^ Six studies used tokenization of words for word-level representation,^47–50^^,^^52^^,^^54^ 1 also tried PV for document-level representation,^48^ and another used the first 40 tokens in a sentence to get sentence-level representation and averaged sentence-level representations to provide document-level representation.^53^ The most common technique for improving representation was token removal, such as removing rare tokens,^47^^,^^50^^,^^52–54^ frequent tokens,^48^^,^^50^^,^^53^^,^^54^ punctuation or special characters,^47^^,^^48^^,^^50^^,^^52^^,^^53^ and stop words.^52^^,^^53^ The most frequently used representation was tf-idf,^48^^,^^52–55^ followed by BoW,^47^^,^^48^^,^^50^^,^^54^ LDA,^47^^,^^52^ GloVe,^49^^,^^50^ ClinicalBERT,^53^^,^^55^ bi-gram,^47^ CBOW,^48^ and PV.^48^ Three studies created a vocabulary of unique terms using BoW,^50^ CBOW,^48^ and tf-idf.^53^ Apostolova and Velez^48^ found that using structured data was inadequate for identifying infection in nursing notes, so they used antibiotic usage and word embeddings to create a labeled dataset of notes with infection, suspected infection, and no infection. Additionally, Horng et al^47^ and Liu et al^50^ listed predictive terms in their models, and Goh et al^52^ provided a list of categories used to classify the top 100 terms. Examples of predictive features are: (1) For sepsis, severe sepsis, or septic shock, Goh et al^52^ classified the top 100-topics into 7 categories: clinical condition or diagnosis, communication between staff, laboratory test order or results, non-clinical condition updates, social relationship information, symptoms, and treatments or medication. (2) Liu et al’s^50^ most predictive NLP terms for the pre-shock versus non-shock state include “tube,” “crrt,” “ards,” “vasopressin,” “portable,” “failure,” “shock,” “sepsis,” and “dl.” (3) Horng et al’s^47^ most predictive terms or topics for having an infection in the ED include “cellulitis,” “sore_throat,” “abscess,” “uti,” “dysuria,” “pneumonia,” “redness_swelling,” “erythema,” “swelling,” “redness, celluititis, left, leg, swelling, area, rle, arm, lle, increased, erythema,” “abcess, buttock, area, drainage, axilla, groin, painful, thigh, left, hx, abcesses, red, boil,” and “cellulitis, abx, pt, iv, infection, po, keflex, antibiotics, leg, treated, started, yesterday.” Whereas the least predictive terms or topics for not having an infection include “motor vehicle crash,” “laceration,” “epistaxis,” “pancreatitis”, “etoh”(ethanol for drunkenness), “etoh, found, vomiting, apparently, drunk, drinking, denies, friends, trauma_neg, triage,” and “watching, tv, sitting, sudden_onset, movie, television, smoked, couch, pt, pot, 5pm, theater.”

ML methods for detecting sepsis using clinical text included: ridge regression,^49^ lasso regression,^54^ logistic regression,^47^^,^^48^^,^^52^ Naïve Bayes (NB),^47^ support vector machines (SVMs),^47^^,^^48^ K-nearest neighbors (KNNs),^48^ random forest (RF),^47^^,^^52^ gradient boosted trees (GBTs),^50–52^^,^^55^ gated recurrent unit (GRU),^50^ and long short-term memory (LSTM).^53^ Although the methods are listed separately, 2 studies combined different ML methods^48^^,^^52^ (see Supplementary Table S4 for details). Ridge and lasso regression are linear regression methods that constrain the model parameters. A linear regression model is represented as $\hat{y}=\beta_{1}x+\beta_{0}$, where $\hat{y}$ is the predicted value, x is the input variable and $\beta_{1}$ and $\beta_{0}$ are model parameters. Model parameters are estimated by minimizing $\sum_{i=1}^{N}{\left(y_{i}-{\hat{y}}_{i}\right)}^{2}$, where $y_{i}$ is the label and N is the number of training samples. In ridge and lasso regression, $\sum_{i=1}^{N}{\left(y_{i}-{\hat{y}}_{i}\right)}^{2}+\lambda \sum_{j=1}^{2}f(\beta_{j})$ is minimized instead, where λ is a hyperparameter that trades-off between fitting the data and model complexity, and $f(z)=z^{2}$ for ridge regression or f(z)=|z| for lasso regression. Logistic regression is a classification method that models $P\left(y|x\right)$, which is the probability of a class y given the feature x. The logistic regression model is defined as $f\left(x\right)=\frac{1}{1+e^{-\left(\beta_{1}x+\beta_{0}\right)}}$. NB is a Bayesian network that eases computation by assuming all input variables are independent given the outcome.^108^ SVM is an extension of a support vector classifier that separates training data points into 2 class regions using a linear decision boundary and classifies new data points based on which region they belong to. To accommodate for non-linearity in the data, SVM enlarges the feature space by applying kernels.^109^ KNNs assume similar data points are close together and use similarity measures to classify new data based on “proximity” to points in the training data.^110^ RF and GBT are ensemble models that use a collection of decision trees to improve the predictive performance of the models. RF classification takes the majority vote of a collection of trees to reduce the decision tree variance.^111^ GBT trains decision trees sequentially so that each tree trains based on information from previously trained trees.^112^^,^^113^ To avoid overfitting, each tree is scaled by a hyperparameter λ, often known as the shrinkage parameter or learning rate that controls the rate the model learns. Recurrent neural networks (RNNs) are a type of NN with recurrent connections and assume that the input data have an ordering, for example, words in a sentence.^114–116^ RNN can be seen as a feed-forward NN with a connection from output to input.^115^ GRU^117^ and LSTM^118^ are improved variations of RNN with gating mechanisms to combat the vanishing gradient problem. The improvements help the models to better model long-term temporal dependencies. To tune hyperparameters, grid-search and Bayesian optimization were used in the studies.^47^^,^^48^^,^^50^^,^^53^^,^^54^ The grid-search method iterates exhaustively through all hyperparameter values within a pre-defined set of values to find the optimal hyperparameter with respect to a validation set. In contrast, the Bayesian optimization method makes informed choices on which values to evaluate using the Bayes formula. The goal of using Bayesian optimization for hyperparameter tuning is to minimize the number of values to evaluate.

**Figure 5.**
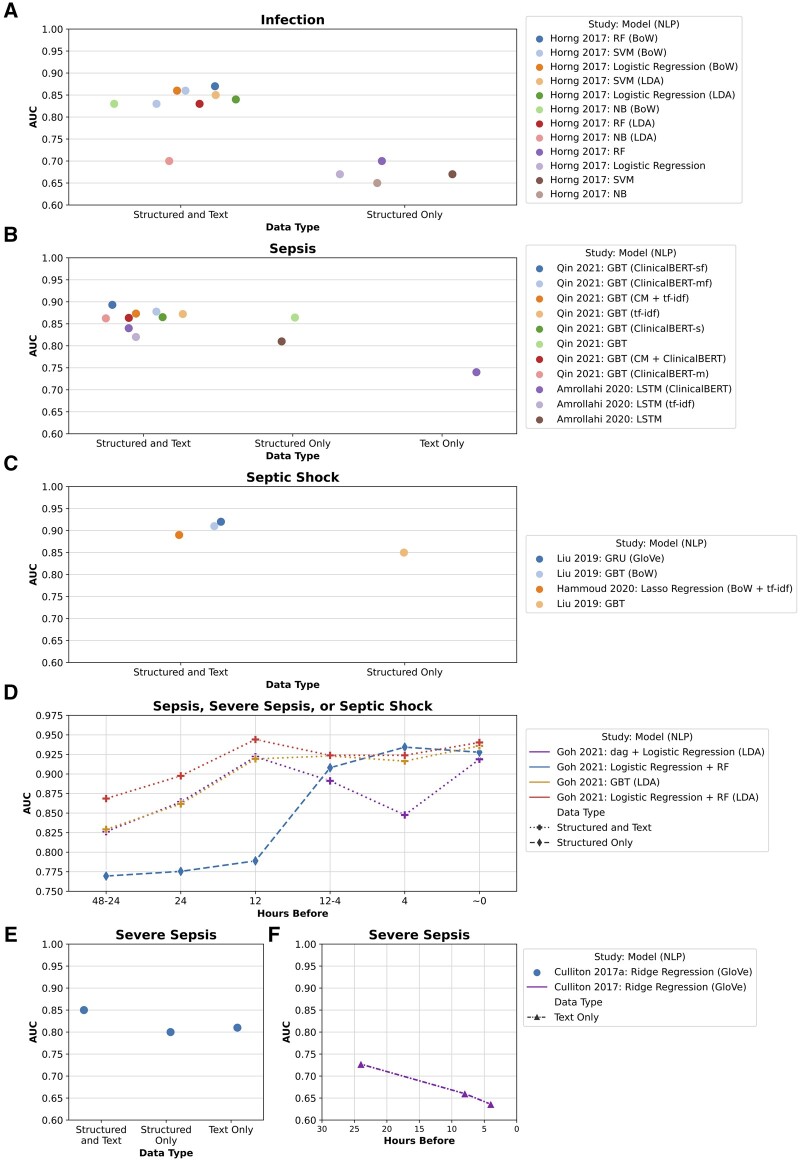
Overview of area under the curve (AUC) values for identification or early detection of infection, sepsis, septic shock, and severe sepsis using different data types (structured data and text, structured data only, and text only).^**∗**^ Each figure contains the study and year, machine learning model,^a^ and natural language processing technique^b^. (A) AUC values for infection identification. Horng et al^47^ 2017: SVM (BoW) has 2 AUC values; 0.86 when using chief complaints and nursing notes and 0.83 when using only chief complaints. (B) AUC values for early sepsis detection. Amrollahi et al^53^ AUC values are from detecting 4 h before sepsis onset, and Qin et al^55^ AUC values are the average from detecting 0 to 6 h before sepsis onset. (C) AUC values for early septic shock detection. Hammoud et al^54^ AUC values are from detecting 30.64 h before septic shock onset, and Liu et al^50^ AUC values are from detecting 6.0 to 7.3 h before septic shock onset. (D) AUC values for early sepsis, severe sepsis, or septic shock detection and sepsis identification in Goh et al.^52^ Different symbols separate data types. (E) AUC values for early septic shock detection for Culliton et al^49^ using results from the test set. (F) AUC values for early septic shock detection for Culliton et al^49^ using results from 3-fold validation. ^**∗**^Disclaimer: AUC values should not be directly compared between studies and different figures for infection, sepsis, severe sepsis, and septic shock. Additionally, the lines connecting points do not indicate AUC values changing over time (Figure  5D and 5F); lines only separate the different methods visually. ^a^Machine learning models: dag: dagging (partition data into disjoint subgroups); GBT: gradient boosted trees; GRU: gated recurrent unit; LSTM: long short-term memory; NB: Naïve Bayes; RF: random forest; SVM: support vector machines. ^b^Natural language processing techniques: BoW: Bag-of-words; ClinicalBERT: Clinical Bidirectional Encoder Representations from Transformers; ClinicalBERT-m: ClinicalBERT from merging all textual features to get embeddings; ClinicalBERT-sf; finetuned ClinicalBERT from concatenating individual embeddings of each textual feature; CM: Amazon Comprehend Medical service for named entity recognition; GloVe: Global Vectors for Word Representation; LDA: Latent Dirichlet Allocation; tf-idf: term frequency-inverse document frequency.

All studies reported evaluation results for different algorithms or data types and almost all reported area under the receiver operating characteristic curve (AUC) values except 1.^48^  Figure  5 shows differences in AUC values for infection (Figure  5A), sepsis (Figure  5B), septic shock (Figure  5C), and severe sepsis (Figure  5E) when using structured data only, text data only, or a combination of structured and text data. Studies that compared their methods for different hours prior to onset are also included (Figure  5D and F), the lines connecting the points are to visually separate the methods and do not indicate changing AUC values over time. This figure compares data type usage and model performance within an individual study; it should not be used to compare AUC values between subfigures and studies because the studies used different cohorts, sepsis definitions, and hours before onset. Additionally, sepsis, severe sepsis, and septic shock have different manifestations.^119^^,^^120^  Table  4 summarizes the best and worst AUC values for each study; a full table with additional evaluation metrics is available in Supplementary Table S4 (number of hours before onset for Amrollahi et al^53^ was confirmed through personal communications with Shamim Nemati on May 27, 2021 and Fatemeh Amrollahi on June 13, 2021). GBT was the most widely used ML method,^50–52^^,^^55^ followed by logistic regression,^47^^,^^48^^,^^52^ SVMs,^47^^,^^48^ RF,^47^^,^^52^ ridge regression,^49^ lasso regression,^54^ NB,^47^ KNNs,^48^ GRU,^50^ and LSTM.^53^ For hyperparameter tuning, 3 studies used the grid-search method^47^^,^^48^^,^^54^ and 2 used the Bayesian optimization method^50^^,^^53^ (hyperparameter tuning was provided by personal communication with Ran Liu on September 7, 2021 and Fatemeh Amrollahi on September 7, 2021). Delahanty et al,^51^ Hammoud et al,^54^ Goh et al,^52^ and Qin et al^55^ compared their algorithm to scoring systems used in clinical practice, such as SIRS,^121^ sequential organ failure assessment (SOFA),^122^ quick SOFA (qSOFA),^123^ modified early warning system (MEWS),^124^ or a targeted real-time early warning score (TREWScore).^78^ In addition, Apostolova and Velez^48^ evaluated their model on a ground truth set with 200 nursing notes that were manually reviewed by a qualified professional, and Goh et al^52^ compared their model with the Rhodes et al^125^ sepsis guidelines used by physicians. Furthermore, Horng et al^47^ performed additional tests on different patient cohorts for error analysis. Although results are difficult to compare directly because of study heterogeneity, most results suggest that utilizing both structured data and text generally results in better performance for sepsis identification and early detection.

**Table 4.** Study outcome overview of best and worst area under the curve values

Study (year)	Hours^a^	Data types^b^		Models^d^ (NLP)^e^	AUC^f^
		DVLMC	T^c^		
Horng et al.^47^ (2017)	Identify	DV- - -	CC + NN	RF (BoW)	0.87
		DV- - -	–	NB	0.65
Apostolova and Velez^48^ (2017)	Identify	- - - - -	NN	SVM (BoW + tf-idf)	–
		- - - - -	NN	Logistic regression + KNN + SVM (PV)	–
Culliton et al.^49^ (2017)	−4	- - - - -	CN	Ridge regression (GloVe)	0.64
	−8	- - - - -	CN	Ridge regression (GloVe)	0.66
	−24	- - - - -	CN	Ridge regression (GloVe)	0.73
	−24^g^	-V- -C	CN	Ridge regression (GloVe)	0.85
		-V- -C	–	Ridge regression (GloVe)	0.80
Delahanty et al.^51^ (2019)	+1	-VL- -	–	GBT	0.93
	+3	-VL- -	–	GBT	0.95
	+6	-VL- -	–	GBT	0.96
	+12	-VL- -	–	GBT	0.97
	+24	-VL- -	–	GBT	0.97
Liu et al.^50^ (2019)	−7	-VLM-	CN	GRU (GloVe)	0.92
	−7.3	-VLM-	CN	GBT (BoW)	0.91
	−6	-VLM-	–	GBT	0.85
Amrollahi et al.^53^ (2020)	−4^h^	-VL- -	PN + NN	LSTM (ClinicalBERT)	0.84
		- - - - -	PN + NN	LSTM (ClinicalBERT)	0.74
Hammoud et al.^54^ (2020)	−30.6	DVL- -	CN	Lasso regression (BoW + tf-idf)	0.89
Goh et al.^52^ (2021)	Identify	DVLM-	PN	Logistic regression + RF (LDA)	0.94
		DVLM-	PN	dag + Logistic regression (LDA)	0.92
	−4	DVLM-	–	Logistic regression + RF	0.93
		DVLM-	PN	dag + Logistic regression (LDA)	0.85
	−6	DVLM-	PN	Logistic regression + RF (LDA)	0.92
		DVLM-	PN	dag + Logistic regression (LDA)	0.89
	−12	DVLM-	PN	Logistic regression + RF (LDA)	0.94
		DVLM-	–	Logistic regression + RF	0.79
	−24	DVLM-	PN	Logistic regression + RF (LDA)	0.90
		DVLM-	–	Logistic regression + RF	0.78
	−48	DVLM-	PN	Logistic regression + RF (LDA)	0.87
		DVLM-	–	Logistic regression + RF	0.77
Qin et al.^55^ (2021)	−6 to 0^i^	-VL- -	CN	GBT (ClinicalBERT-sf)	0.89^i^
		-VL- -	–	GBT (ClinicalBERT-m)	0.86^i^

a Hours: Identify: not detecting hours before or after; –: hours before; +: hours after an event.

b Data types: D: demographics; V: vitals; L: laboratory; M: medications; C: codes; T: text; -‘s position in DVLMC indicates which is not used.

c Text data types: CC: chief complaints; CN: various types of clinical notes; NN: nursing notes; PN: physician notes; –: no notes.

d Machine learning models: dag: dagging (partition data into disjoint subgroups); GBT: gradient boosted trees; GRU: gated recurrent unit; KNN: K-nearest neighbors; LSTM: long short-term memory; NB: Naïve Bayes; RF: random forest; SVM: support vector machines.

e Natural language processing (NLP) techniques: BoW: Bag-of-words; ClinicalBERT: Clinical Bidirectional Encoder Representations from Transformers; ClinicalBERT-m: ClinicalBERT from merging all textual features to get embeddings; ClinicalBERT-sf: finetuned ClinicalBERT from concatenating individual embeddings of each textual feature; GloVe: Global Vectors for Word Representation; LDA: Latent Dirichlet Allocation; PV: paragraph vectors; tf-idf: term frequency-inverse document frequency.

f Area under the curve (AUC). Apostolova and Velez^48^ did not provide metrics for AUC.

g Culliton et al^49^ performed 2 experiments, these results are from using a test set instead of 3-fold validation.

h Number of hours before onset for Amrollahi et al^53^ was confirmed through personal communications (with Shamim Nemati on May 27, 2021 and Fatemeh Amrollahi on June 13, 2021).

i Qin et al^55^ AUC values are an average from 0 to 6 h before sepsis, not the specified hours.

## DISCUSSION

### Identification, early detection, prediction, and method transferability

Nine studies utilized clinical text for sepsis identification, early detection, or prediction. As all identified studies focus on the identification or early detection of sepsis within a fixed time frame, this indicates much work is still needed before sepsis prediction can use text from complete patient histories. Studies from this review focus mainly on the ICU and ED, and the addition of continuous measurements of vital signs for sepsis makes generalizability to the ward units limited. However, Culliton et al^49^ was successful in detecting sepsis early utilizing only the text from EHR clinical notes, which is a promising approach for all inpatients. Additionally, Horng et al^47^ showed that their ML model performed on subsets of specific patient cohorts like pneumonia or urinary tract infection. The different ML methods and NLP techniques from each study may be applicable for different retrospective cohort or case–control studies. Though the studies have varying sepsis definitions, cohorts, ML methods, and NLP techniques, overall, they show that using clinical text and structured data can improve sepsis identification and early detection. Unstructured clinical text predicts sepsis 48–12 h before onset, while structured data predicts sepsis closer to onset (<12 h before).

### Sepsis definition impact

In ML, many studies rely heavily on sepsis definitions and ICD-codes to identify patient cohort datasets for sepsis studies.^9^^,^^11^^,^^13^ Among changing sepsis definitions over time are the 2001 Angus Sepsis ICD-9 abstraction criteria,^79^ 2012 Surviving Sepsis Campaign Guidelines,^126^ 2016 Sepsis-3 consensus definition,^1^ and 2017 Rhee’s modified Sepsis-3 definition.^80^ Although a consensus sepsis definition exists,^1^ not all definition elements will be present in a sepsis patient because sepsis is a very heterogeneous syndrome^127^ and the infection site is difficult to identify correctly.^128^ Many patients with sepsis are often misdiagnosed with other diseases such as respiratory failure^129^ and pneumonia.^129^^,^^130^ In practice, hospitals also have varying sepsis coding methods.^131–135^ As the sepsis definitions change, studies also tend to use the most current definition in their study. A recent study that used different sepsis definitions to generate patient cohorts found significant heterogeneous characteristics and clinical outcomes between cohorts.^136^ Similarly, previous work by Liu et al^137^ demonstrated that using different infection criteria resulted in a different number of patients and slightly different outcomes. Similar to how changes in the definition and varying coding methods can affect sepsis mortality outcomes,^138^ the sepsis definition and codes used in ML studies will likely change the outcome, results, and reporting methods. Thus, future studies should acknowledge that sepsis is a syndrome and clearly characterize each sign of sepsis to reflect the heterogeneity in the definition.

### Suggestions for future studies

Predicting sepsis earlier than 12 h prior to sepsis onset can reduce treatment delays and improve patient outcomes.^3^^,^^4^ Because predictions 48–12 h before sepsis onset appear to rely more on clinical text than structured data, additional NLP techniques should be considered for future ML studies. Additionally, since the sepsis definition used will change the cohort, this indicates opportunities to expand the cohort. Like Apostolova and Velez,^48^ who determined their cohort by finding notes describing the use of antibiotics. It should be possible to determine cohorts by using notes describing infection signs (eg, fever, hypotension, or deterioration in mental status), indicators of diseases that sepsis is misdiagnosed with (eg, pulmonary embolism, adrenal insufficiency, diabetic ketoacidosis, pancreatitis, anaphylaxis, bowel obstruction, hypovolemia, colitis, or vasculitis), or medication effect and toxin ingestion, overdose, or withdrawal.^139^ NLP methods from infectious diseases known to trigger sepsis can be incorporated to extract infection signs and symptoms from the text for determining potential sepsis signs, patient groups, and risk factors. For instance, many sepsis patients are often admitted with pneumonia, and there are several studies about identifying pneumonia from radiology reports using NLP.^23^^,^^140^^,^^141^ Additionally, heterogeneous sepsis signs or symptoms might be identified by utilizing NLP features for detecting healthcare-associated infections risk patterns^59^ or infectious symptoms.^142^ Information from other NLP related reviews about using clinical notes can also be applied, such as: challenges to consider,^16^ clinical information extraction tools and methods,^18^ methods to overcome the need for annotated data,^22^ different embedding techniques,^143^^,^^144^ sources of labeled corpora,^143^ transferability of methods,^145^ and processing and analyzing symptoms.^146^ Moreover, heterogeneous or infectious diseases, with overlapping signs and symptoms of other diseases, can utilize similar sepsis ML and NLP methods to improve detection. The identified studies did not utilize complete patient history data. Thus, future research utilizing complete patient history data can study if sepsis risk can be predicted earlier than 48 h by incorporating sepsis risk factors, such as comorbidities,^7^ chronic diseases,^147^ patient trajectories,^148^ or prior infection incidents.^149^

### Limitations

This review has several limitations. The narrow scope of including only studies about utilizing clinical text for sepsis detection or prediction could have missed studies that use other types of text for sepsis detection or prediction. For example, search terms did not include “early warning system,” “feature extraction,” and “topic modeling.” Additionally, search terms did not include possible sources of infection for sepsis, such as bloodstream infection, catheter-associated infection, pneumonia, and postoperative surgical complications. Further, the sensitivity to detect sepsis in text, structured data, or the combined data from these will depend on the timestamps these data recordings have in the EHR. These timestamps may vary depending on the data used to inform the study or the different systems implemented at different hospitals. The articles identified in this review had a homogenous choice of structured data (ie, demographics, vital signs, and laboratory measurements). Of those, laboratory test results have the largest time lag, around 1–2 h to obtain the blood test results.^150^ Thus, the good performance of text to detect sepsis in these articles are unlikely explained fully by the time lag between measurement and recording of the structured data. This review thus shows that it is possible to detect sepsis early using text, with or without the addition of structured data.

## CONCLUSION

Many studies about sepsis detection exist, but very few studies utilize clinical text. Heterogeneous study characteristics made it difficult to compare results; however, the consensus from most studies was that combining structured data with clinical text improves identification and early detection of sepsis. There is a need to utilize the unstructured text in EHR data to create early detection models for sepsis. The lack of utilizing the complete patient history in early prediction models for sepsis is an opportunity for future ML and NLP studies.

## FUNDING

Financial support for this study was provided by the Computational Sepsis Mining and Modelling project through the Norwegian University of Science and Technology Health Strategic Area.

## AUTHOR CONTRIBUTIONS

MYY and ØN conceptualized the study and design with substantial clinical insight from LTG. MYY conducted the literature search and initial analysis, LTG verified results, and ØN resolved discrepancies. All authors participated in data analysis and interpretation. MYY drafted the manuscript, which LTG and ØN critically revised.

## SUPPLEMENTARY MATERIAL

Supplementary material is available at *Journal of the American Medical Informatics Association* online.

## Supplementary Material

ocab236_Supplementary_Data
