# Supplementary material for: Sepsis prediction, early detection, and identification using clinical text for machine learning: a systematic review
Source: J Am Med Inform Assoc. 2021 Dec 13;29(3):559–75. doi: 10.1093/jamia/ocab236 (PMC8800516; doi:10.1093/jamia/ocab236)
Supplement: ocab236_Supplementary_Data [file ocab236_supplementary_data.pdf]

Supplementary Materials

for

"Sepsis Prediction, Early Detection and Identification Using Clinical Text for Machine Learning: A Systematic Review"

Authors: Melissa Y. Yan, MBI[1], Lise Tuset Gustad, PhD[2,3] and Øystein Nytrø, PhD[1]

Affiliations:

[1] Department of Computer Science, Norwegian University of Science and Technology, Trondheim, Norway

[2] Department of Circulation and Medical Imaging, Norwegian University of Science and Technology, Trondheim, Norway

[3] Department of Medicine, Levanger Hospital, Clinic of Medicine and rehabilitation, Nord-Trøndelag Hospital Trust, Levanger, Norway

* To whom correspondence should be addressed, Email: melissa.yan@ntnu.no

There were nine included studies in the literature review. Horng et al.[1] and Apostolova and Velez[2] focused on sepsis infection identification. Culliton et al.[3], Delahanty et al.[4], Liu et al.[5], Amrollahi et al.[6], Hammoud et al.[7] and Qin et al.[8] focused on early detection of sepsis,[4,6,8] severe sepsis[3] or septic shock.[5,7] And Goh et al.[9] considered both identification and early detection for sepsis, severe sepsis and septic shock.

Supplementary Table S1: Search Strategies for Each Database Based on Defined Search Terms

Database	Database Type*	Search Terms†				Search Query
		1	2	3	4	
PubMed	CL	TA	AF	AF	AF	(("sepsis"[Title/Abstract] OR "septic shock"[Title/Abstract] OR "systemic inflammatory response syndrome"[Title/Abstract] OR "SIRS"[Title/Abstract]) AND ("natural language processing"[All Fields] OR "natural language processing"[MeSH Terms] OR "machine learning"[All Fields] OR "machine learning"[MeSH Terms] OR "artificial intelligence"[All Fields] OR "artificial intelligence"[MeSH Terms] OR "unstructured data"[All Fields] OR "unstructured text"[All Fields] OR "clinical note"[All Fields] OR "clinical notes"[All Fields] OR "clinical text"[All Fields] OR "free-text"[All Fields] OR "free-text"[All Fields] OR "record text"[All Fields] OR "narrative"[All Fields] OR "narratives"[All Fields]) AND ("predict"[All Fields] OR "predictabilities"[All Fields] OR "predictability"[All Fields] OR "predictable"[All Fields] OR "predictably"[All Fields] OR "predicted"[All Fields] OR "predicting"[All Fields] OR "prediction"[All Fields] OR "predictions"[All Fields] OR "predictive"[All Fields] OR "predictively"[All Fields] OR "predictiveness"[All Fields] OR "predictives"[All Fields] OR "predictivities"[All Fields] OR "predictivity"[All Fields] OR "predicts"[All Fields] OR ("detect"[All Fields] OR "detectabilities"[All Fields] OR "detectability"[All Fields] OR "detectable"[All Fields] OR "detectables"[All Fields] OR "detectably"[All Fields] OR "detected"[All Fields] OR "detectible"[All Fields] OR "detecting"[All Fields] OR "detection"[All Fields] OR "detections"[All Fields] OR "detects"[All Fields]) OR ("identifiable"[All Fields] OR "identifiably"[All Fields] OR "identifie"[All Fields] OR "identified"[All Fields] OR "identifier"[All Fields] OR "identifiers"[All Fields] OR "identifies"[All Fields] OR "identify"[All Fields] OR "identifying"[All Fields]) OR ("identifed"[All Fields] OR "identification, psychological"[MeSH Terms] OR ("identification"[All Fields] AND "psychological"[All Fields]) OR "psychological identification"[All Fields] OR "identification"[All Fields] OR "identifications"[All Fields]) OR ("diagnosable"[All Fields] OR "diagnosi"[All Fields] OR "diagnosis"[MeSH Terms] OR "diagnosis"[All Fields] OR "diagnose"[All Fields] OR "diagnosed"[All Fields] OR "diagnoses"[All Fields] OR "diagnosing"[All Fields] OR "diagnosis"[MeSH Subheading]) OR ("prognosis"[MeSH Terms] OR "prognosis"[All Fields] OR "prognoses"[All Fields]) OR ("recognisable"[All Fields] OR "recognise"[All Fields] OR "recognised"[All Fields] OR "recognises"[All Fields] OR "recognising"[All Fields] OR

						"recognize"[All Fields] OR "recognized"[All Fields] OR "recognizes"[All Fields] OR "recognizing"[All Fields]) OR ("disease progression"[MeSH Terms] OR ("disease"[All Fields] AND "progression"[All Fields]) OR "disease progression"[All Fields] OR "progression"[All Fields] OR "progress"[All Fields] OR "progressed"[All Fields] OR "progresses"[All Fields] OR "progressing"[All Fields] OR "progressions"[All Fields] OR "progressive"[All Fields] OR "progressively"[All Fields] OR "progressives"[All Fields]) OR ("develop"[All Fields] OR "develope"[All Fields] OR "developed"[All Fields] OR "developer"[All Fields] OR "developer s"[All Fields] OR "developers"[All Fields] OR "developing"[All Fields] OR "developments"[All Fields] OR "develops"[All Fields] OR "growth and development"[MeSH Subheading] OR ("growth"[All Fields] AND "development"[All Fields]) OR "growth and development"[All Fields] OR "development"[All Fields]) OR ("age of onset"[MeSH Terms] OR ("age"[All Fields] AND "onset"[All Fields]) OR "age of onset"[All Fields] OR "onset"[All Fields] OR "onsets"[All Fields] OR "onsetting"[All Fields]))) NOT ("mice"[MeSH Terms] OR "mice"[All Fields] OR ("mice"[MeSH Terms] OR "mice"[All Fields] OR "mouse"[All Fields] OR "mouse s"[All Fields] OR "mouses"[All Fields]) OR ("rats"[MeSH Terms] OR "rats"[All Fields] OR "rat"[All Fields]) OR ("rats"[MeSH Terms] OR "rats"[All Fields]) OR ("animals"[MeSH Terms:noexp] OR "animal"[All Fields]) OR ("animals"[MeSH Terms:noexp] OR "animals"[All Fields]) OR ("nonhuman"[All Fields] OR "nonhumans"[All Fields]))
Scopus	CL	TA	AF	AF	AF	(((TITLE-ABS-KEY (sepsis) OR TITLE-ABS-KEY ("septic shock") OR TITLE-ABS-KEY ("systemic inflammatory response syndrome")) AND (ALL ("natural language processing") OR ALL ("machine learning") OR ALL ("artificial intelligence") OR ALL ({unstructured data}) OR ALL ({unstructured text}) OR ALL ({clinical note}) OR ALL ({clinical notes}) OR ALL ({clinical text}) OR ALL ({free-text}) OR ALL ({free text}) OR ALL ({record text}) OR ALL ({narrative}) OR ALL ({narratives})) AND ((predict*) OR (detect*) OR (identif*) OR (diagnos*) OR (prognos*) OR (recog*) OR (progres*) OR (develop*) OR (onset)))) AND NOT (ALL (mice) OR ALL (mouse) OR ALL (rat*) OR ALL (animal*) OR ALL (nonhuman*))
ACM DL	CS	AF	-	-	-	[All: sepsis] OR [All: “septic shock”] OR [All: “systemic inflammatory response syndrome”]
dblp	CS	AF	-	-	-	3 separate searches:<br>1. “sepsis”<br>2. “septic shock”<br>3. “systemic inflammatory response syndrome”

IEEE Xplore	CS	AF	-	-	-	(“All Metadata”:sepsis) OR (“All Metadata”:septic shock) OR (“All Metadata”:systemic inflammatory response syndrome)

* Database types: CL = Clinical and CS = computer science

† Search terms usage in: TA = Title OR Abstract, AF = All fields or - = not used. Defined Search Terms: 1 = sepsis OR “septic shock” OR “systemic inflammatory response syndrome”; 2 = “natural language processing” OR “machine learning” OR “artificial intelligence” OR “unstructured data” OR “unstructured text” OR “clinical note” OR “clinical notes” OR “clinical text” OR “free-text” OR “free text” OR “record text” OR “narrative” OR “narratives”; 3 = detect* OR identif* OR recog* OR diagnos* OR predict* OR prognos* OR develop* OR onset*; 4 = “mice” OR “mouse” OR “rat” OR “rats” OR “animal” OR “nonhuman”. Search Terms 1, 2 and 3 are for inclusion and search term 4 is for exclusion.

ACM DL: Association for Computing Machinery Digital Library; dblp: dblp computer science bibliography; IEEE Xplore: Institute of Electrical and Electronics Engineers Xplore digital library

Supplementary Table S2. Study Variables and Sepsis Definitions

Study	Variables	Full Sepsis Definition Used
Horng et al.[1] (2017)	Age, gender, acuity, systolic blood pressure, diastolic blood pressure, heart rate, pain scale, respiratory rate, oxygen saturation, temperature, chief complaint, nursing assessment	Angus Sepsis ICD-9-CM abstraction criteria[10]:<br>• Bacterial or fungal infections codes: 001, 002, 003, 004, 005, 008, 009, 010, 011, 012, 013, 014, 015, 016, 017, 018, 020, 021, 022, 023, 024, 025, 026, 027, 030, 031, 032, 033, 034, 035, 036, 037, 038, 039, 040, 041, 090, 091, 092, 093, 094, 095, 096, 097, 098, 100, 101, 102, 103, 104, 110, 111, 112, 114, 115, 116, 117, 118, 320, 322, 324, 325, 420, 421, 451, 461, 462, 463, 464, 465, 481, 482, 485, 486, 491.21, 494, 510, 513, 540, 541, 542, 562.01, 562.03, 562.11, 562.13, 566, 567, 569.5, 569.83, 572.0, 572.1, 575.0, 590, 597, 599.0, 601, 614, 615, 616, 681, 682, 683, 686, 711.0, 730, 790.7, 996.6, 998.5, 999.3<br>• Acute organ dysfunction classification codes: 785.5, 458, 96.7, 348.3, 293, 348.1, 287.4, 287.5, 286.9, 286.6, 570, 573.4, 584
Apostolova and Velez[2] (2017)	None	Notes describing patient taking or being prescribed antibiotics to treat infection.
Culliton et al.[3] (2017) *	29 physiological related variables and clinical notes*<br>• heart rate H,M,S<br>• respiratory rate H,M,S<br>• systolic blood pressure M,S<br>• temperature H,L,M,N,S<br>• mean arterial blood pressure M,S<br>• white blood count H,L,M,N,S	Modified Baystate clinical definition of severe sepsis (8 structured variables) and severe sepsis ICD codes:<br>• Activated partial thromboplastin time<br>• Bilirubin<br>• Creatinine<br>• Normalized clotting time<br>• Lactate<br>• Mean arterial pressure

	• creatinine [M]<br>• lactate [H,M,S]<br>• platelets [M,S]<br>• bilirubin [M]<br>• activated partial thromboplastin time [M]<br>• normalized clotting time [M]<br>• Over 100 types of notes, but mostly:<br>• history-and-physical notes<br>• progress reports	• Platelet count<br>• Systolic blood pressure<br>Sepsis adult inpatient, exhibits two of three symptoms[*]:<br>• Temperature > 100.9 or < 96.8<br>• Pulse Rate > 90<br>• Respiratory Rate > 20<br>• White blood count > 12,000 or <4,000 or > 10% band<br>Severe sepsis adult inpatient, has sepsis and 1 of the following[*]:<br>• Systolic Blood Pressure < 90 or Mean Arterial Pressure < 65 or drop in Systolic Blood Pressure by 40 points<br>• Creatinine > 2.0 or Urine output < 0.5ml/kg/hr for 2 hours<br>• Bilirubin > 2 mg/dl (34.2 mmol/L)<br>• Platelet count < 100,000<br>• Clotting time > 1.5 or activated partial thromboplastin > 60<br>• Lactate > 4 mmol/L (18.0 mg/dl)<br>• Acute Respiratory Failure- documented need for ventilation (mechanical or non-mechanical)
Delahanty et al.[4] (2019)	Items evaluated for inclusion, not all were retained in the final model:<br>• demographic (age)<br>• medication (dobutamine, dopamine, epinephrine, norepinephrine)<br>• engineered feature (systolic blood pressure x age, hour/systolic blood pressure, hour/systolic blood pressure x age)<br>• laboratory result (anion gap, albumin, alkaline phosphatase, aspartate aminotransferase, bands, bilirubin, BUN, calcium, creatinine, glomular filtration rate, glucose, lactic acid,	Rhee's modified Sepsis-3 definition[11]

	lymphocyte, monocyte, neutrophil, platelet, white blood cell count)<br>• neurological evaluation (Glasgow coma scale)<br>• structured nursing documentation (supplemental oxygen, ventilator, altered mental status)<br>• text-keyword from emergency department chief complaint ("abscess", "acute", "altered", "bacteremia", "cellulitis", "cystitis", "diabetes", "failure", "lactic", "leukocytosis", "PNA", "pneumonia", "pyelonephritis", "respiratory", "sepsis", "septic", "urosepsis", "UTI")<br>• vitals (heart rate, mean arterial pressure, respiratory rate, systolic blood pressure, oxygen saturation, temperature)<br>• physiological (weight, height, BMI)	
Liu et al.[5] (2019)	• 28 physiological variables from previous study[12]:<br>o heart rate, respiratory rate, temperature, SBP, DBP, mean BP, CVP, PaO2, FiO2, GCS, bilirubin, platelets, creatinine, lactate, BUN, arterial pH, WBC, PaCO2, hemoglobin, hematocrit, potassium, epinephrine, dopamine, dobutamine, norepinephrine, phenylephrine, vasopressin, urine output<br>• Over 2 million clinical notes	Sepsis-3 definition[13]
Amrollahi et al.[6] (2020)	• 40 physiological and clinical features from other studies[14,15]:<br>o Demographics (age, gender, unit1 ICU identifier, unit2 ICU identifier, Hours between hospital and ICU admission, hours since ICU admission)<br>o Vital signs (heart rate, pulse oximetry, temperature, systolic BP, mean arterial pressure, diastolic BP, respiration rate, end tidal carbon dioxide)<br>o Laboratory values (measure of excess bicarbonate, bicarbonate, fraction of inspired oxygen, pH, partial pressure of carbon dioxide from arterial blood, oxygen saturation from arterial blood, aspartate transaminase,	Sepsis-3 definition[13]

	blood urea nitrogen, alkaline phosphatase, calcium, chloride, creatinine, direct bilirubin, serum glucose, lactic acid, magnesium, phosphate, potassium, total bilirubin, troponin I, hematocrit, hemoglobin, partial thromboplastin time, leukocyte count (WBC), fibrinogen concentration, platelet count)<br>• Physician and nursing notes	
Hammoud et al.[7] (2020)	26 vital, clinical and laboratory measurements from Henry et al.[16]:<br>• age<br>• vital signs (respiratory rate, systolic blood pressure, diastolic blood pressure, mean arterial pressure, heart rate, SpO2, FiO2, Glasgow Coma Score, Riker sedation-agitation score, urine output, fluid input)<br>• laboratory test results (blood urea nitrogen, creatinine, white blood cell count, hematocrit, hemoglobin, potassium, bicarbonate, arterial pH, PaO2, PaCO2)<br>• clinical history (HIV, chronic liver disease, chronic heart failure, chronic organ insufficiency, immunocompromised state, hematologic malignancy, current type of care unit)<br><br>All notes from MIMIC-II except discharge summaries, such as but not limited to nursing and respiratory therapist progress notes	Sepsis definition based on Henry et al.[16]:<br>• Infection suspicion based on Angus Sepsis ICD-9-CM abstraction criteria [10] or clinical note mentioning sepsis or septic shock<br>• SIRS criteria and sepsis-related organ dysfunction criteria from Surviving Sepsis Campaign guidelines [17]<br>• SIRS = any two SIRS criteria simultaneously present<br>• Sepsis = SIRS + infection suspicion<br>• Severe sepsis = sepsis + sepsis-related organ dysfunction<br>• Septic shock = severe sepsis + have hypotension + received adequate fluid resuscitation
Goh et al.[9] (2021)	• Patient information: age, gender<br>• Vital signs (blood pressure, heart rate, temperature, oxygen saturation, respiratory rate)<br>• Investigations (total white cell, various culture results (urine, blood, general, wound, stool, molecular, fluid, respiratory, cerebrospinal fluid, fungus smear, tissue, ear, genital, sterility testing, tip culture, appearance, stain results or any real-time polymerase chain reaction), lactate, high-sensitivity C-reactive protein, procalcitonin, arterial blood gas)	ICU admission with an ICD-10 code for sepsis, severe sepsis or sepsis shock:<br>• Sepsis: A40.0, A40.1, A40.8, A40.9, A41.2, A41.0, A41.0Z16, A41.1, A40.3, A41.4, A41.50, A41.3, A41.51, A41.52, A41.53, A41.59, A41.81, A41.89, A41.9<br>• Severe Sepsis: R65.20, R65.21, R65.10, R65.11

	• treatment (use of vasopressor, use of antibiotics, clinical notes)	
Qin et al.[8] (2021)	• Vital signs (heart rate, systolic blood pressure, diastolic blood pressure, mean arterial pressure, respiratory rate, temperature, oxygen saturation, blood glucose levels, partial pressure of carbon dioxide<br>• Nursing notes, physician notes, radiology notes and respiratory notes	PhysioNet Challenge restrictive Sepsis-3 definition[8,18]:<br>• Infection suspicion: Earlier timestamp of either IV antibiotics given first with blood culture obtained within 24-hours, or blood cultures obtained first with antibiotics ordered within 72-hours and administered for at least 72-consecutive hours.<br>• SOFA: Occurrence of two-point increase in SOFA score within 24-hours.<br>• Sepsis: Earliest timestamp of infection suspicion or SOFA when both occur within a 24-hour period

ICD: International Classification of Diseases; ICD-9 CM: ICD Clinical Modification, 9th revision; ICD-10: ICD 10th revision; IV: intravenous; MIMIC-II: Multiparameter Intelligent Monitoring in Intensive Care II database; SIRS: Systemic Inflammatory Response Syndrome; SOFA: Sequential Organ Failure Assessment

[*]For Culliton et al.[3], the structured variables used were provided through personal communications (with Steve Gallant on June 4, 2021), and the severe sepsis and sepsis definitions were also provided through personal communications (with Steve Gallant on September 6, 2021). The 12 structured variables selected for severe sepsis prediction are listed in the table and the resulting 29 structured variables used are: ^{H} = abnormal high readings, ^{L} = abnormal low readings, ^{M} = mean, ^{N} = normal readings and ^{S} = standard deviation.

Supplementary Table S3. Types of Variables Used to Create Models in Studies

Category	Name	Study									
		**Horng et al.**[1]	**Apostolova and Velez**[2]	**Culliton et al.**[3]*	**Delahanty et al.**[4]†	**Liu et al.**[5]‡	**Amrollahi et al.**[6]	**Hammoud et al.**[7]§	**Goh et al.**[9]	**Qin et al.**[8]	**# Studies using variable**
Demographics	age	x			x		x	x	x		5
	gender	x					x		x		3
	administrative identifier for MICU						x				1
	administrative identifier for SICU						x				1
	Hours between hospital admittance and ICU admittance						x				1
	ICU length-of-stay						x				1
Vital signs	heart rate	x		x[H,M,S]	x	x	x	x	x	x	8
	respiratory rate	x		x[H,M,S]	x[†]	x	x	x		x	7
	systolic blood pressure (SBP)	x		x[M,S]	x[†]	x	x	x		x	7
	temperature	x		x[H,L,M,N,S]	x[†]	x	x		x	x	7
	oxygen saturation (SpO2)	x			x		x	x	x	x	6
	diastolic blood pressure (DBP)	x				x	x	x		x	5
	mean arterial pressure (MAP)			x[M,S]	x		x	x		x	5
	blood pressure								x		1
	central venous pressure (CVP)					x					1
	end tidal carbon dioxide						x				1
	mean blood pressure					x					1
	Riker sedation-agitation score							x			1
Neurological evaluation	Glasgow coma scale (GCS)				x	x		x			3
Laboratory results	white blood count (WBC)			x[H,L,M,N,S]	x[†]	x	x	x	x		6
	creatinine			x[M]	x[†]	x	x	x			5

	blood urea nitrogen (BUN)				x	x	x	x			4
	lactate			x[H,M,S]		x	x		x		4
	platelets			x[M,S]	x	x	x				4
	bilirubin			x[M]	x	x					3
	Fraction of inspired oxygen (FiO2)					x	x	x			3
	hematocrit					x	x	x			3
	hemoglobin					x	x	x			3
	partial pressure of carbon dioxide from arterial blood (PaCO2)					x	x	x		x	4
	potassium					x	x	x			3
	alkaline phosphatase				x		x				2
	arterial pH					x		x			2
	bicarbonate						x	x			2
	calcium				x		x				2
	glucose				x†		x			x	3
	PaO2					x		x			2
	activated partial thromboplastin time			x[M]							1
	albumin				x†						1
	anion gap				x						1
	arterial blood gas								x		1
	aspartate aminotransferase				x						1
	aspartate transaminase (AST)						x				1
	bands				x						1
	bilirubin direct						x				1
	bilirubin total						x				1
	chloride						x				1
	fibrinogen						x				1
	glomular filtration rate				x						1
	high-sensitivity C-reactive protein								x		1
	lactic acid				x†						1
	lymphocyte				x						1
	magnesium						x				1
	measure of excess bicarbonate						x				1
	monocyte				x						1
	neutrophil				x†						1
	normalized clotting time (INR)			x[M]							1

	oxygen saturation from arterial blood (SaO2)						x				1
	partial thromboplastin time						x				1
	pH						x				1
	phosphate						x				1
	procalcitonin								x		1
	troponin I						x				1
	various cultures								x		1
Medicine	epinephrine				x	x		x			3
	dobutamine				x	x					2
	dopamine				x	x					2
	norepinephrine				x	x					2
	phenylephrine					x					1
Treatment	antibiotics use								x		1
	vasopressor use								x		1
Other	urine output					x		x			2
	acuity	x									1
	fluid input							x			1
	pain scale	x									1
	vasopressin					x					1
Physiological	body mass index				x						1
	height				x						1
	weight				x						1
Structured nursing documentation	altered mental status				x						1
	supplemental oxygen				x						1
	ventilator				x						1
History	chronic heart failure							x			1
	chronic liver disease							x			1
	chronic organ insufficiency							x			1
	current type of care unit							x			1
	hematologic malignancy							x			1
	HIV							x			1
	immunocompromised state							x			1
Engineered feature	systolic blood pressure X age				x						1
	shock index (= pulse rate / systolic blood pressure)				x						1

	shock index X age				x						1
Clinical Text	nursing notes	x	x			x	x	x		x	6
	clinical notes - other or not specified			x		x		x			3
	physician notes					x	x		x	x	4
	chief complaint	x			x						2
	electrocardiogram (ECG)					x		x			2
	pharmacy reports					x		x			2
	radiology notes					x		x		x	3
	respiratory therapist progress notes					x		x		x	3
	consultation notes					x					1
	discharge summaries					x					1
	echocardiogram							x			1
	history-and-physical notes			x							1
	progress reports			x							1
Keyword	text-keyword from emergency department chief complaint ("abscess", "acute", "altered", "bacteremia", "cellulitis", "cystitis", "diabetes", "failure", "lactic", "leukocytosis", "PNA", "pneumonia", "pyelonephritis", "respiratory", "sepsis", "septic", "urosepsis", "UTI")				x						1

* Structured variables used in Culliton et al.[3] (provided by Steve Gallant on June 4, 2021). The 12 structured variables selected for severe sepsis prediction are listed in the table and the resulting 29 structured variables used are: ^{H} = abnormal high readings, ^{L} = abnormal low readings, ^{M} = mean, ^{N} = normal readings and ^{S} = standard deviation.

† variable used in final model of Delahanty et al.[4].

‡ Types of notes and usage for Liu et al.[5] (provided by Ran Liu on June 2, 2021). Discharge summaries were used to build a vocabulary of unique terms and unlikely used for prediction.

§ Types of notes for Hammoud et al.[7] (provided by Ibrahim Hammoud on May 29, 2021).

Supplementary Table S4. Complete Study Outcome Evaluations

Study (Year)	Hours*	Data Types†		Models§ (NLP)¶	Evaluation Metrics#					
		DVLMC	T‡		AUC**	Precision (Positive predictive value (PPV))	Recall (Sensitivity; True positive rate (TPR))	Specificity (True negative rate (TNR))	False positive rate (FPR)	F1-score
Horng et al.[1] (2017)	identify	DV---	-	SVM	0.670	0.220	0.560	0.680	0.320	0.316
		DV---	CC	SVM (BoW)	0.830	0.320	0.750	0.750	0.250	0.449
		DV---	CC + NN	SVM (BoW)	0.860	0.380	0.780	0.790	0.210	0.511
		DV---	CC + NN	SVM (LDA)	0.850	0.340	0.800	0.750	0.250	0.477
		DV---	-	Logistic Regression	0.670	-	-	-	-	-
		DV---	CC + NN	Logistic Regression (BoW)	0.860	-	-	-	-	-
		DV---	CC + NN	Logistic Regression (LDA)	0.840	-	-	-	-	-
		DV---	-	NB	0.650	-	-	-	-	-
		DV---	CC + NN	NB (BoW)	0.830	-	-	-	-	-
		DV---	CC + NN	NB (LDA)	0.700	-	-	-	-	-
		DV---	-	RF	0.700	-	-	-	-	-
		DV---	CC + NN	RF (BoW)	0.870	-	-	-	-	-
		DV---	CC + NN	RF (LDA)	0.830	-	-	-	-	-
Apostolova and Velez[2] (2017)	identify	-----	NN	SVM (BoW + tf-idf) ††	-	0.931	0.990	-	-	0.960
		-----	NN	Logistic Regression + KNN + SVM (PV)	-	0.630	0.770	-	-	0.693
Culliton et al.[3] (2017)	– 4	-----	CN	Ridge Regression (GloVe)	0.636	-	-	-	-	-
	– 8	-----	CN	Ridge Regression (GloVe)	0.660	-	-	-	-	-
	– 24	-----	CN	Ridge Regression (GloVe)	0.727	-	-	-	-	-
	– 24‡‡	-----	CN	Ridge Regression (GloVe)	0.810	-	-	-	-	-
		-V--C	-	Ridge Regression (GloVe)	0.800	-	-	-	-	-

		-V--C	CN	Ridge Regression (GloVe)	0.850	-	-	-	-	-
Delahanty et al.[4] (2019)	+ 1	-VL--	-	GBT	0.930	0.276	0.677	0.964	0.036	0.392
	+ 3	-VL--	-	GBT	0.950	0.300	0.721	0.966	0.034	0.424
	+ 6	-VL--	-	GBT	0.960	0.319	0.749	0.968	0.032	0.447
	+ 12	-VL--	-	GBT	0.970	0.308	0.793	0.964	0.036	0.444
	+ 24	-VL--	-	GBT	0.970	0.288	0.846	0.958	0.042	0.430
Liu et al.[5] (2019)	– 6	-VLM-	-	GBT	0.850	0.430	0.760	0.790	0.210	0.549
	– 7.3	-VLM-	CN	GBT (BoW)	0.910	0.460	0.860	0.790	0.210	0.599
	– 7	-VLM-	CN	GRU (GloVe)	0.920	0.490	0.840	0.820	0.180	0.619
Amrollahi et al.[6] (2020)	– 4[§§]	-----	NN + PN	LSTM (ClinicalBERT)	0.740	-	0.850	0.460	0.540	-
		-VL--	-	LSTM	0.810	-	0.850	0.630	0.370	-
		-VL--	NN + PN	LSTM (tf-idf)	0.820	-	0.850	0.630	0.370	-
		-VL--	NN + PN	LSTM (ClinicalBERT)	0.840	-	0.850	0.670	0.330	-
Hammoud et al.[7] (2020)	– 30.64	DVL--	CN	Lasso Regression (BoW + tf-idf)	0.890	-	-	0.670	0.330	-
Goh et al.[9] (2021)[¶¶]	identify	DVLM-	-	Logistic Regression + RF	0.928	-	0.868	0.863	0.137	-
		DVLM-	PN	Logistic Regression + RF (LDA)	0.940	0.854	0.885	0.869	0.131	0.869
		DVLM-	PN	dag + Logistic Regression (LDA)	0.919	-	-	-	-	-
		DVLM-	PN	GBT (LDA)	0.936	-	-	-	-	-
	– 4	DVLM-	-	Logistic Regression + RF	0.934	-	0.865	0.861	0.139	-
		DVLM-	PN	Logistic Regression + RF (LDA)	0.924	0.813	0.865	0.802	0.199	0.838
		DVLM-	PN	dag + Logistic Regression (LDA)	0.848	-	-	-	-	-
		DVLM-	PN	GBT (LDA)	0.917	-	-	-	-	-
	– 6	DVLM-	-	Logistic Regression + RF	0.908	-	0.864	0.817	0.183	-
		DVLM-	PN	Logistic Regression + RF (LDA)	0.924	0.820	0.881	0.807	0.193	0.850
		DVLM-	PN	dag + Logistic Regression (LDA)	0.891	-	-	-	-	-
		DVLM-	PN	GBT (LDA)	0.923	-	-	-	-	-
	– 12	DVLM-	-	Logistic Regression + RF	0.789	-	0.757	0.727	0.273	-

		DVLM-	PN	Logistic Regression + RF (LDA)	0.944	0.874	0.872	0.874	0.126	0.873
		DVLM-	PN	dag + Logistic Regression (LDA)	0.922	-	-	-	-	-
		DVLM-	PN	GBT (LDA)	0.919	-	-	-	-	-
	– 24	DVLM-	-	Logistic Regression + RF	0.776	-	0.778	0.715	0.285	-
		DVLM-	PN	Logistic Regression + RF (LDA)	0.898	0.800	0.809	0.797	0.203	0.804
		DVLM-	PN	dag + Logistic Regression (LDA)	0.864	-	-	-	-	-
		DVLM-	PN	GBT (LDA)	0.862	-	-	-	-	-
	– 48	DVLM-	-	Logistic Regression + RF	0.770	-	0.714	0.687	0.313	-
		DVLM-	PN	Logistic Regression + RF (LDA)	0.869	0.770	0.782	0.767	0.233	0.776
		DVLM-	PN	dag + Logistic Regression (LDA)	0.826	-	-	-	-	-
		DVLM-	PN	GBT (LDA)	0.829	-	-	-	-	-
Qin et al.[8] (2021) ##	– 0	-VL--	-	GBT	0.864	-	0.830	0.725	0.275	-
		-VL--	CN	GBT (tf-idf)	0.872	-	0.854	0.754	0.246	-
		-VL--	CN	GBT (CM + tf-idf)	0.873	-	0.848	0.741	0.259	-
		-VL--	CN	GBT (CM + ClinicalBERT)	0.863	-	0.860	0.709	0.291	-
		-VL--	CN	GBT (ClinicalBERT-s)	0.865	-	0.843	0.717	0.283	-
		-VL--	CN	GBT (ClinicalBERT-m)	0.862	-	0.844	0.735	0.265	-
		-VL--	CN	GBT (f-ClinicalBERT-s)	0.893	-	0.866	0.766	0.235	-
		-VL--	CN	GBT (f-ClinicalBERT-m)	0.878	-	0.872	0.751	0.249	-
	– 1	-VL--	-	GBT	0.864	-	0.819	0.725	0.275	-
		-VL--	CN	GBT (tf-idf)	0.872	-	0.844	0.754	0.246	-
		-VL--	CN	GBT (CM + tf-idf)	0.873	-	0.836	0.741	0.259	-
		-VL--	CN	GBT (CM + ClinicalBERT)	0.863	-	0.849	0.709	0.291	-
		-VL--	CN	GBT (ClinicalBERT-s)	0.865	-	0.833	0.717	0.283	-
		-VL--	CN	GBT (ClinicalBERT-m)	0.862	-	0.834	0.735	0.265	-
		-VL--	CN	GBT (f-ClinicalBERT-s)	0.893	-	0.858	0.766	0.235	-
		-VL--	CN	GBT (f-ClinicalBERT-m)	0.878	-	0.862	0.751	0.249	-
	– 2	-VL--	-	GBT	0.864	-	0.810	0.725	0.275	-

		-VL--	CN	GBT (tf-idf)	0.872	-	0.838	0.754	0.246	-
		-VL--	CN	GBT (CM + tf-idf)	0.873	-	0.832	0.741	0.259	-
		-VL--	CN	GBT (CM + ClinicalBERT)	0.863	-	0.840	0.709	0.291	-
		-VL--	CN	GBT (ClinicalBERT-s)	0.865	-	0.822	0.717	0.283	-
		-VL--	CN	GBT (ClinicalBERT-m)	0.862	-	0.825	0.735	0.265	-
		-VL--	CN	GBT (f-ClinicalBERT-s)	0.893	-	0.850	0.766	0.235	-
		-VL--	CN	GBT (f-ClinicalBERT-m)	0.878	-	0.851	0.751	0.249	-
	– 3	-VL--	-	GBT	0.864	-	0.793	0.725	0.275	-
		-VL--	CN	GBT (tf-idf)	0.872	-	0.827	0.754	0.246	-
		-VL--	CN	GBT (CM + tf-idf)	0.873	-	0.821	0.741	0.259	-
		-VL--	CN	GBT (CM + ClinicalBERT)	0.863	-	0.823	0.709	0.291	-
		-VL--	CN	GBT (ClinicalBERT-s)	0.865	-	0.811	0.717	0.283	-
		-VL--	CN	GBT (ClinicalBERT-m)	0.862	-	0.810	0.735	0.265	-
		-VL--	CN	GBT (f-ClinicalBERT-s)	0.893	-	0.840	0.766	0.235	-
		-VL--	CN	GBT (f-ClinicalBERT-m)	0.878	-	0.841	0.751	0.249	-
	– 4	-VL--	-	GBT	0.864	-	0.771	0.725	0.275	-
		-VL--	CN	GBT (tf-idf)	0.872	-	0.803	0.754	0.246	-
		-VL--	CN	GBT (CM + tf-idf)	0.873	-	0.799	0.741	0.259	-
		-VL--	CN	GBT (CM + ClinicalBERT)	0.863	-	0.807	0.709	0.291	-
		-VL--	CN	GBT (ClinicalBERT-s)	0.865	-	0.790	0.717	0.283	-
		-VL--	CN	GBT (ClinicalBERT-m)	0.862	-	0.784	0.735	0.265	-
		-VL--	CN	GBT (f-ClinicalBERT-s)	0.893	-	0.811	0.766	0.235	-
		-VL--	CN	GBT (f-ClinicalBERT-m)	0.878	-	0.813	0.751	0.249	-
	– 5	-VL--	-	GBT	0.864	-	0.746	0.725	0.275	-
		-VL--	CN	GBT (tf-idf)	0.872	-	0.769	0.754	0.246	-
		-VL--	CN	GBT (CM + tf-idf)	0.873	-	0.767	0.741	0.259	-
		-VL--	CN	GBT (CM + ClinicalBERT)	0.863	-	0.774	0.709	0.291	-
		-VL--	CN	GBT (ClinicalBERT-s)	0.865	-	0.769	0.717	0.283	-

		-VL--	CN	GBT (ClinicalBERT-m)	0.862	-	0.746	0.735	0.265	-
		-VL--	CN	GBT (f-ClinicalBERT-s)	0.893	-	0.787	0.766	0.235	-
		-VL--	CN	GBT (f-ClinicalBERT-m)	0.878	-	0.787	0.751	0.249	-
	– 6	-VL--	-	GBT	0.864	-	0.701	0.725	0.275	-
		-VL--	CN	GBT (tf-idf)	0.872	-	0.729	0.754	0.246	-
		-VL--	CN	GBT (CM + tf-idf)	0.873	-	0.723	0.741	0.259	-
		-VL--	CN	GBT (CM + ClinicalBERT)	0.863	-	0.731	0.709	0.291	-
		-VL--	CN	GBT (ClinicalBERT-s)	0.865	-	0.726	0.717	0.283	-
		-VL--	CN	GBT (ClinicalBERT-m)	0.862	-	0.706	0.735	0.265	-
		-VL--	CN	GBT (f-ClinicalBERT-s)	0.893	-	0.736	0.766	0.235	-
		-VL--	CN	GBT (f-ClinicalBERT-m)	0.878	-	0.743	0.751	0.249	-

[*] Hours: identify = not detecting hours before or after, – = hours before and + = hours after an event

[†] Data types: D = demographics, V = vitals, L: lab, M = medications, C = codes, T = text and - position in DVLMC indicates which is not used.

[‡] Text data types: CC = chief complaints, CN = various types of clinical notes, NN = nursing notes, PN = physician notes and - = no notes.

[§] Machine learning models: dag = dagging (partition data into disjoint subgroups), GBT = gradient boosted trees, GRU = gated recurrent network, KNN = K-nearest neighbors, LSTM = long-short term memory, NB = Naïve Bayes, RF = random forest and SVM = support vector machines.

[¶] Natural language processing (NLP) techniques: BoW = Bag-of-words, ClinicalBERT = Clinical Bidirectional Encoder Representations from Transformers, ClinicalBERT-m = ClinicalBERT from merging all textual features to get embeddings, ClinicalBERT-sf = finetuned ClinicalBERT from concatenating individual embeddings of each textual feature, CM = Amazon Comprehend Medical service for named entity recognition, GloVe = Global Vectors for Word Representation, LDA = Latent Dirichlet Allocation, PV = paragraph vectors and tf-idf = term frequency-inverse document frequency.

[#] Evaluation metrics: - = values not provided or calculable with given data and blue elements are values calculated from provided values.

[**] Area under the curve (AUC).

†† Apostolova and Velez[2] results from evaluating the model on a ground truth set with 200 nursing notes which were manually reviewed by a qualified professional.

‡‡ Culliton et al.[3] performed two experiments, these results are from using a test set instead of 3-fold validation.

§§ Number of hours before onset for Amrollahi et al.[6] was confirmed through personal communications (with Shamim Nemati on May 27, 2021 and Fatemeh Amrollahi on June 13, 2021).

¶¶ Goh et al.[9] AUC values are from using the synthetic minority over-sampling (SMOTE) technique to adjust sepsis cases to be up to 50% of the overall sample and were obtained from their supplemental Peer Review File.

Qin et al.[8] AUC values are an average from 0-6 hours before sepsis, not the specified hours.

References

1 Horng S, Sontag DA, Halpern Y, *et al.* Creating an automated trigger for sepsis clinical decision support at emergency department triage using machine learning. *PLoS One* 2017;**12**:e0174708. doi:10.1371/journal.pone.0174708

2 Apostolova E, Velez T. Toward Automated Early Sepsis Alerting: Identifying Infection Patients from Nursing Notes. In: *BioNLP 2017*. Association for Computational Linguistics 2017. 257–62. doi:10.18653/v1/W17-2332

3 Culliton P, Levinson M, Ehresman A, *et al.* Predicting Severe Sepsis Using Text from the Electronic Health Record. In: *Workshop on Machine Learning For Health at the Conference on Neural Information Processing Systems (NIPS ML4H 2017)*. 2017. http://arxiv.org/abs/1711.11536

4 Delahanty RJ, Alvarez J, Flynn LM, *et al.* Development and Evaluation of a Machine Learning Model for the Early Identification of Patients at Risk for Sepsis. *Ann Emerg Med* 2019;**73**:334–44. doi:10.1016/j.annemergmed.2018.11.036

5 Liu R, Greenstein JL, Sarma SV, *et al.* Natural Language Processing of Clinical Notes for Improved Early Prediction of Septic Shock in the ICU. *Conf Proc IEEE Eng Med Biol Soc* 2019;**2019**:6103–8. doi:10.1109/EMBC.2019.8857819

6 Amrollahi F, Shashikumar SP, Razmi F, *et al.* Contextual embeddings from clinical notes improves prediction of sepsis. *AMIA Annu Symp Proc* 2020;**2020**:197–202.https://www.ncbi.nlm.nih.gov/pubmed/33936391

7 Hammoud I, Ramakrishnan IV, Henry M, *et al.* Multimodal early septic shock prediction model using lasso regression with decaying response. In: *2020 IEEE International Conference on Healthcare Informatics (ICHI)*. IEEE 2020. doi:10.1109/ichi48887.2020.9374377

8 Qin F, Madan V, Ratan U, *et al.* Improving early sepsis prediction with multi modal learning. arXiv [cs.CL]. 2021.http://arxiv.org/abs/2107.11094

9 Goh KH, Wang L, Yeow AYK, *et al.* Artificial intelligence in sepsis early prediction and diagnosis using unstructured data in healthcare. *Nat Commun* 2021;**12**:711. doi:10.1038/s41467-021-20910-4

10 Angus DC, Linde-Zwirble WT, Lidicker J, *et al.* Epidemiology of severe sepsis in the United States: analysis of incidence, outcome, and associated costs of care. *Crit Care Med* 2001;**29**:1303–10. doi:10.1097/00003246-200107000-00002

11 Rhee C, Dantes R, Epstein L, *et al.* Incidence and Trends of Sepsis in US Hospitals Using Clinical vs Claims Data, 2009-2014. *JAMA* 2017;**318**:1241–9. doi:10.1001/jama.2017.13836

12 Liu R, Greenstein JL, Granite SJ, *et al.* Data-driven discovery of a novel sepsis pre-shock state predicts impending septic shock in the ICU. *Sci Rep* 2019;**9**:6145. doi:10.1038/s41598-019-42637-5

13 Singer M, Deutschman CS, Seymour CW, *et al.* The Third International Consensus Definitions for Sepsis and Septic Shock (Sepsis-3). *JAMA* 2016;**315**:801–10. doi:10.1001/jama.2016.0287

14 Reyna M, Clifford G. Early prediction of sepsis from clinical data -- the PhysioNet Computing in Cardiology Challenge 2019. 2019. doi:10.13026/V64V-D857

15 Reyna MA, Josef CS, Jeter R, *et al.* Early Prediction of Sepsis From Clinical Data: The PhysioNet/Computing in Cardiology Challenge 2019. *Crit Care Med* 2020;**48**:210–7. doi:10.1097/CCM.0000000000004145

16 Henry KE, Hager DN, Pronovost PJ, *et al.* A targeted real-time early warning score (TREWScore) for septic shock. *Sci Transl Med* 2015;**7**:299ra122. doi:10.1126/scitranslmed.aab3719

17 Dellinger RP, Levy MM, Rhodes A, *et al.* Surviving sepsis campaign: international guidelines for management of severe sepsis and septic shock: 2012. *Crit Care Med* 2013;**41**:580–637. doi:10.1097/CCM.0b013e31827e83af

18 Reyna MA, Josef C, Seyedi S, *et al.* Early Prediction of Sepsis from Clinical Data: the PhysioNet/Computing in Cardiology Challenge 2019. In: *2019 Computing in Cardiology (CinC)*. 2019. Page 1-Page 4. doi:10.23919/CinC49843.2019.9005736
